# Two linked *TBXT* (brachyury) gene polymorphisms are associated with the tailless phenotype in fat‐rumped sheep

**DOI:** 10.1111/age.12852

**Published:** 2019-09-02

**Authors:** J. Han, M. Yang, T. Guo, C. Niu, J. Liu, Y. Yue, C. Yuan, B. Yang

**Affiliations:** ^1^ Lanzhou Institute of Husbandry and Pharmaceutical Sciences Chinese Academy of Agricultural Sciences Lanzhou 730050 China; ^2^ College of Animal Science and Technology Shihezi University Shihezi 832000 China; ^3^ Key Laboratory of Animal (Poultry) Genetics Breeding and Reproduction Ministry of Agriculture Beijing 100083 China; ^4^ International Livestock Research Institute (ILRI) PO Box 30709 Nairobi 00100 Kenya

**Keywords:** caudal vertebrae, causative mutations, Kazakh sheep, tail type, T-box gene

## Abstract

*T‐box transcription factor T* (*TBXT*), encoding the brachyury protein, is an embryonic nuclear transcription factor involved in mesoderm formation and differentiation. Previous studies indicate that *TBXT* mutations are responsible for the tailless or short‐tailed phenotype of many vertebrates. To verify whether the tailless phenotype in fat‐rumped sheep is associated with *TBXT* mutations, exon 2 of the *TBXT* gene for 301 individuals belonging to 13 Chinese and Iranian sheep breeds was directly sequenced. Meanwhile, 380 samples were used to detect the genotypes of the candidate variations by mapping to their reads databases in the Sequence Read Archive repository of GenBank. The results showed that one missense mutation, c.334G>T (GGG>TGG) with a completely linked synonymous variant c.333G>C (CCG>CCC) was found to be associated with the ‘tailless’ characteristic in typical fat‐rumped sheep breeds. The c.334G>T transversion led to the conversion of glycine to tryptophan at the 112th amino acid in the T‐box domain of the brachyury protein. In addition, crossbreeding experiments for long‐tailed and tailless sheep showed that CT/CT allele of nucleotides (nt) 333 and 334, a recessive mutation, would cause sheep tails to be shorter, suggesting that these two linked variants at nucleotides 333 and 334 in *TBXT* are probably causative mutations responsible for the tailless phenotype in sheep.

Tails exhibit a wide range of functions, such as balance, locomotion and communication, in different animals. Tail shortening or loss may have occurred independently multiple times during evolution (e.g. in cats, dogs and monkeys; Buckingham *et al*. 2013). Generally, tail length is directly proportional to the number of caudal vertebrae, and variation in tail length both within and among species is a ubiquitous phenomenon. Sheep have a long history of domestication with tails varying in size and shape, including short‐, long‐, thin‐ and fat‐tailed as well as fat‐rumped phenotypes, across different breeds and/or geographic regions. Typically, tail length of sheep can be classified into three categories: ‘tailless’, which describes a tail with a small number of caudal vertebrae hidden in the rump's fat; ‘short tail’, which means a tail with 7–16 caudal vertebrae (approximately 14–24 cm long) and ending above the hock joint; and ‘long tail’, which represents a tail with 18–24 caudal vertebrae and reaching to the hock joint or below. Long tails incur many disadvantages in practice because they have a significant influence on mating and normal locomotion. Routine tail docking makes it easier for sheep farmers to implement management practices, but it is forbidden in many countries owing to concern for animal welfare (Indrebo *et al*. 2008). Large fat tails have lost their importance in recent decades; however, the short, thin tail is desirable in sheep breeding. The typical fat‐rumped sheep born with a ‘tailless’ phenotype could serve as a natural model to study the inheritance pattern of tail phenotypes in sheep. Numerous studies have identified many genes affecting tail development, such as the *PTF1A* (Vlangos *et al*. 2013), *SNAIL1* (Thisse *et al*. 1993), *TBXT* (Halpern *et al*. 1993; Schulte‐Merker *et al*. 1994), *HOXB13* (Economides *et al*. 2003), *CDX2* (van de Ven *et al*. 2011) and *HES7* (Xu *et al*. 2016) genes. Most of these genes are members of the Hox family of genes and T‐box transcription factors. The T‐box family is an ancient group that appears to play a critical role in vertebral development in all animal species. In particular, the *TBXT* gene is necessary for maintaining axial extension, which plays a key role in the development of caudal vertebrae in mouse embryos (Gluecksohn‐Schoenheimer 1938). Genetic variants in *TBXT* have been investigated to explore their association with tailless or very short‐tailed phenotypes in many vertebrates. In particular, mutations in the T‐box region of *TBXT* have been identified to affect the development of the tail vertebrae in cats, dogs and cattle (Hytonen *et al*. 2009; Buckingham *et al*. 2013; Kromik *et al*. 2015a, 2015b). In cats, multiple *TBXT* variant alleles are associated with short‐tailed phenotypes (Buckingham *et al*. 2013). The c.189C>G mutation in *TBXT* is correlated with the short‐tail phenotype, and the GG homozygote is lethal in dogs (Haworth *et al*. 2001; Hytonen *et al*. 2009). The spontaneous mutation c.196A>G in the taurine cattle *TBXT* gene leads to vertebral and spinal dysplasia in Holstein cattle (Kromik *et al*. 2015a, 2015b). Recently, the c.334G>T mutation in *TBXT* was reported to contribute to the short‐tail phenotype of Hulunbuir short‐tailed sheep (Zhi *et al*. 2018). According to these findings, it is likely that the *TBXT* gene could be considered as a candidate gene regulating the tail types in sheep. Therefore, we collected samples from sheep with different tail types and used their sequencing data to determine whether the mutations in the coding sequence of the *TBXT* gene are associated with the ‘tailless’ phenotype in fat‐rumped sheep.

To compare the tail lengths of different sheep breeds, three lambs from Alpine Merino (long‐tailed), Tibetan (short) and Kazakh sheep (tailless) were selected (Fig. 1a). Radiographic examination was performed to measure the number of caudal vertebrae after removing the tails of lambs at slaughter. We also observed the number of caudal vertebrae in adult Kazakh sheep by carefully removing the adipose tissue and muscle after boiling the tails of slaughtered animals. Blood samples of 301 individuals belonging to 13 breeds with different tail types were collected from China and Iran and were used for DNA extraction (Table S1). We also collected blood samples from their crossbred offspring, including six individuals from the F1 generation from Tibetan sheep crossbred with Kazakh sheep, six F1 individuals from Texel sheep crossed with Kazakh sheep and 18 F2 individuals from their F1 offspring backcrossed with Kazakh sheep (Table S2). In order to validate these variations in expanded samples originating from a broader geographic area, Gutiérrez‐Gil's (2017) method was introduced into this study. We used a 70‐bp sequence covering the two mutations (CGCTGGAAGTACGTGAACGGGGAGTGGGTGCCGGGGGGCAAGCCGGAGCCGCAGGCGCCCAGCTGCG) as the query sequence and performed blastn against reads databases in the Sequence Read Archive (SRA); nearly 1200 individual sheep from the bioproject of the International Sheep Genomics Consortium and another 25 bioprojects were analyzed (Table S1). Only the alignments with a range greater than 40 bp and identities greater than 92% of the results were considered to be credible.

**Figure 1:**
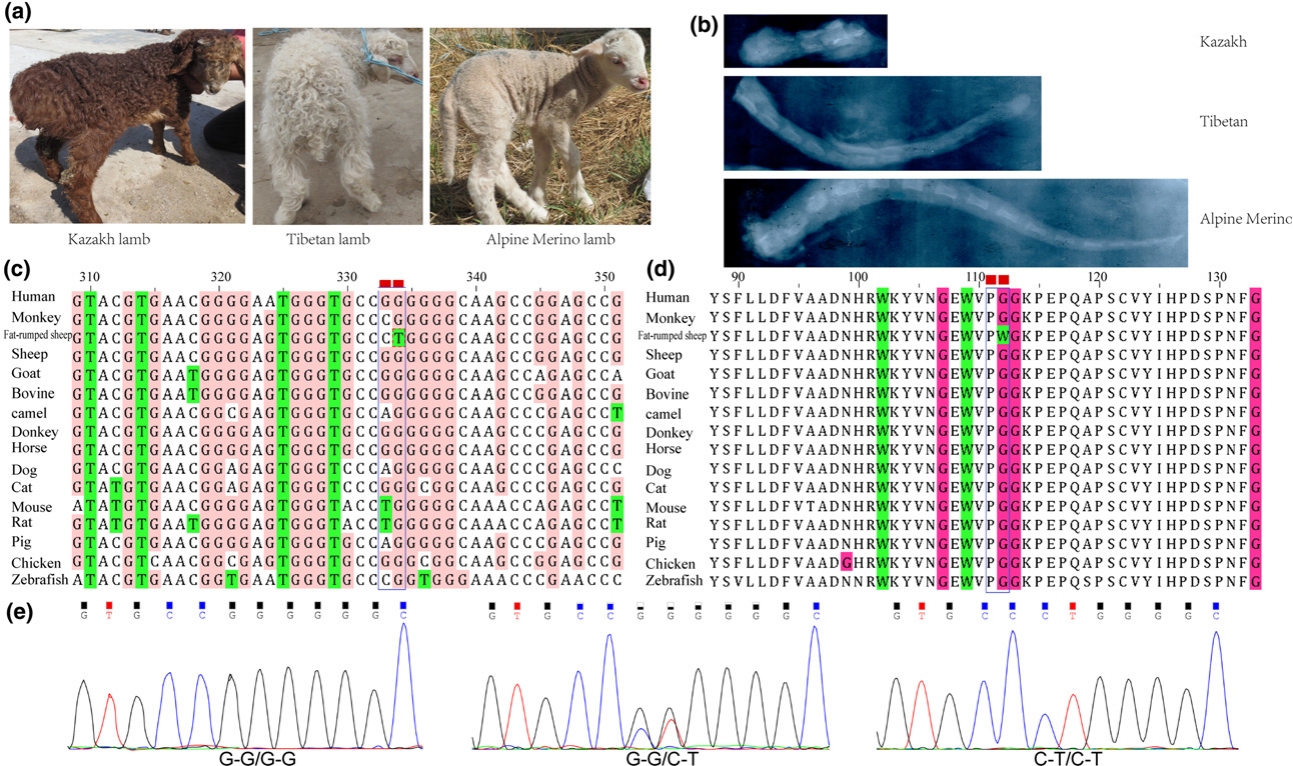
(a) Tail phenotypes of Kazakh, Tibetan and Alpine Merino from China. (b) X‐ray photographs of the caudal vertebrates in the three sheep breeds. (c) Sequence alignment of c.[333G>C;334G>T] in *TBXT* gene across 15 vertebrates. (d) Brachyury protein/homolog encompassing the variant fat‐rumped sheep amino acid position 112 across 15 vertebrates. (e) Electropherogram showing a part of the exon 2 nucleotide sequences near nucleotides 333 and 334 of the sheep *TBXT* gene at genome position.

The quality of genomic DNA was measured by NanoDrop 2000 (Thermo Scientific) after extraction from the blood. Eight pairs of primers were then designed to amplify the nine exons of the *TBXT* gene in sheep using ENSOARG00000004863.1 in Ensemble as a reference sequence. All the primers and optimal annealing temperatures are indicated in Table S3. Exons of 10 Alpine Merino, 10 Tibetan and 10 Kazakh sheep individuals were amplified and sequenced to find the candidate SNPs in *TBXT*. The obtained sequences were further processed using the Chromas MFC application for SNP discovery. The results suggested that 31 mutations were present in these breeds, and all the variations were submitted to European Variation Archive under the project no. PRJEB31336 (Table S4). Among them, a missense mutation c.334G>T (8:87804589, GGG>TGG) and a synonymous mutation c.333G>C (8:87804590, CCG>CCC) in exon 2 were of interest (Table S4; Fig. 1e). To further characterize these two linked *TBXT* variants and their association with tail types of sheep, 681 sample sequences, obtained both from SRA databases and PCR sequencing data, containing these two sites from 32 breeds/strains with different tail types were analyzed. Specifically, 380 individual sheep in the SRA databases, from across the world, were believed to contain valid reads for these two candidate variations after sequence alignment of their reads databases was performed, and 301 of these individuals belonged to 13 Chinese and Iranian sheep breeds with PCR sequences of exon 2, which also targets these two linked gene polymorphisms (Table S1). Association between the genotype c.[333G>C;334G>T] alleles and tail phenotype was tested by chi‐square analysis. In addition, exon 2 of the crossbred offspring was sequenced to verify if the mutations were under autosomal dominant inheritance. Furthermore, sequence homology across species was analyzed using amino acid sequences of the brachyury protein from 15 typical vertebrates downloaded from UniProt (http://www.uniprot.org/) and mRNA sequences collected from NCBI (http://www.ncbi.nlm.nih.gov/), including sheep, goat, cattle, horse, donkey, camel, pig, human, dog, cat, monkey, mouse, rat, chicken and zebrafish. Sequence analysis and multiple alignments were carried out using clustalw and jalview 2.10.2 (Waterhouse *et al*. 2009).

Radiographic examination showed that four to five very short caudal vertebrae were observed in the Kazakh sheep (Fig. S1), which was far fewer than in Tibetan and Alpine Merino sheep (Fig. 1b). The vertebrae were hidden in the rump fat. Therefore, the tail phenotype of the fat‐rumped sheep seems to be ‘tailless’. Furthermore, we also found that sheep tail types were associated with the genotype of c.[333G>C;334G>T] alleles. In particular, the genotype CT/CT of *TBXT*:c.[333G>C;334G>T] was present only in the fat‐rumped sheep, fat‐rump‐like sheep and semi‐fat‐tailed sheep, and it was the main genotype for Chinese fat‐rumped sheep. It was absent in long‐tailed and short‐tailed breeds, in which the dominant genotype was GG/GG (Table S1). The results supported our hypothesis that the genotype CT/CT for the TBXT:c.[333G>C;334G>T SNPs is significantly associated with the ‘tailless’ characteristic (*P* < 2.2 × 10^−16^), indicating that these two linked causative mutations in the *TBXT* gene could influence the numbers of caudal vertebrae in sheep.

Pfam domain analysis suggested that amino acid sites for nucleotides (nt) 333 and 334 are located at residues 111 and 112 and within the T‐box domain of the brachyury protein, where the DNA target binding region is located, and it can activate gene transcription. As a result, *TBXT*:c.333G>C is a synonymous mutation and *TBXT*:c.334G>T will lead to a glycine (G) to tryptophan (W) substitution at residue 112. To estimate the conservation of nt 333 and 334 among different species, mRNA and their amino acid sequences of *TBXT* in fat‐rumped sheep were compared with that of other vertebrates. Finally, it turned out that the sites were conserved in vertebrates (Fig. 1c, d), which suggested that the mutations might influence the DNA binding in the T‐box domain. Further functional studies will be required to validate the influence of the nt 333 and 334 on *TBXT*.

**Figure 2:**
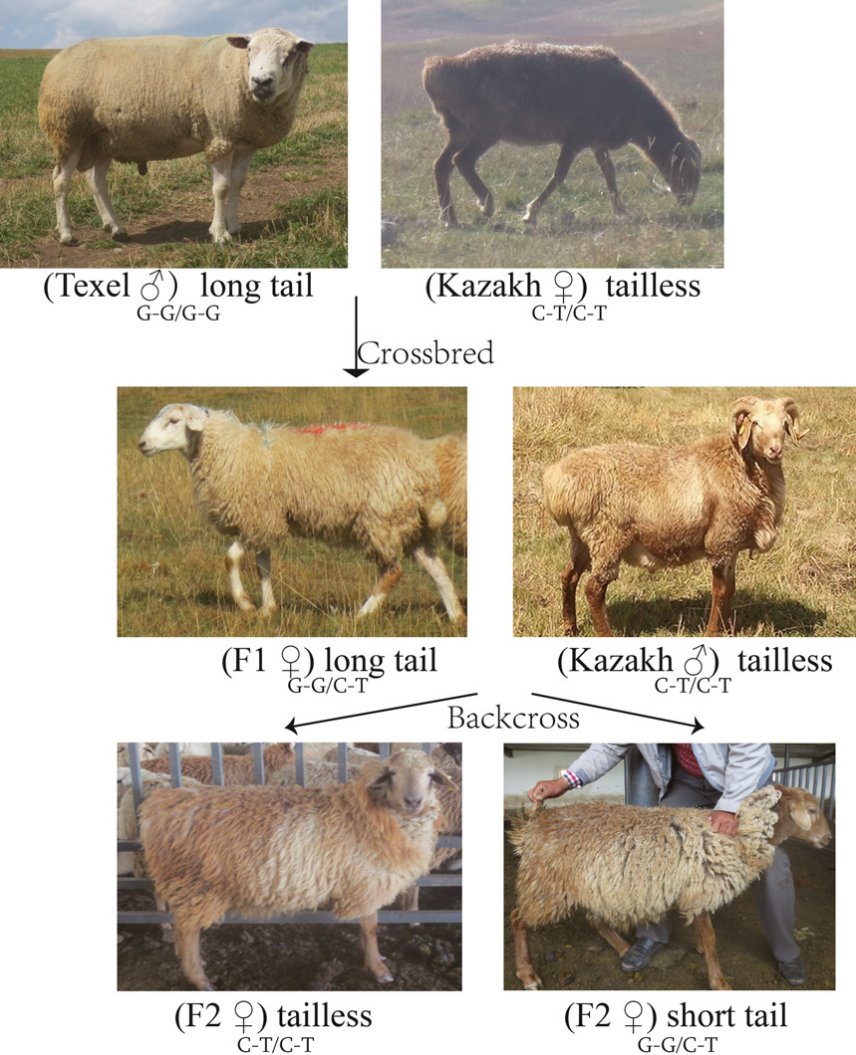
Tail phenotype of F2 offspring from F1 ewes (Texel crossbred with Kazakh sheep) backcrossed with Kazakh rams.

Furthermore, to verify whether *TBXT*:c.[333G>C;334G>T] are dominant or recessive mutations, the tail type and genotype of the mutations from 12 crossbred F1 offspring and 18 F2 offspring were measured. As expected, all the F1 hybrids were heterozygous GG/CT mutations at nt 333 and 334 of the *TBXT* gene and had a normal or long‐tail characteristic similar to their parents’ tail type (Table S2; Fig. 2). The results revealed that 10 F2 offspring were heterozygous GG/CT with short tails and eight F2 offspring were CT/CT with a tailless phenotype (Table S2, Fig. 2), indicating that the GG haplotype presented a single dominance gene inheritance and CT/CT could be a recessive genotype for the ‘tailless’ phenotype in fat‐rumped sheep.

The *TBXT* gene is a tissue‐specific transcription factor expressed in the notochord and primitive streak during embryonic development. Therefore, the *TBXT* gene is essential for trunk/tail primary mesoderm formation and migration from the primitive streak, which drives axis elongation (Zhu *et al*. 2016). The *TBXT* gene is expressed only in the early stages of notochord development, and this gene can regulate the transforming growth factor and Wnt signalling pathway during vertebrate development (Hayashi *et al*. 2016). Mutations in the *TBXT* gene are associated with skeletal defects in heterozygotes, but individuals homozygous for the mutations exhibit severe developmental disorders (Meisler 1997). Short‐tailed dogs were all heterozygous for the mutation in the *TBXT* gene (Haworth *et al*. 2001; Indrebo *et al*. 2008) and the heterozygous *TBXT* gene mutations are a common cause of taillessness or short tails in cats (Buckingham *et al*. 2013). Short‐tailed mice were also observed to be heterozygous for the *TBXT* mutations (Wu *et al*. 2010). Unfortunately, the homozygous *TBXT* mutations were found to be lethal in early fetal life of these animals. Nevertheless, the homozygous CT/CT genotype at nt 333 and 334 was the dominant type in the Chinese fat‐rumped sheep of this present study and was also detected both in fat‐rump‐like Mehraban and semi‐fat‐tailed Dalagh sheep in Iran. The homozygous *TBXT* mutations represent only restrained development in the caudal vertebrae in these sheep, which suggests that the c.[333G>C;334G>T] SNPs could be an advantagous mutation for fat accumulation transferred from the tail to the hips for these fat‐rumped sheep, may be enabling them to store more fat to cope with periods of food shortage. Similarly, a search of the SRA database under project no. PRJNA386449 revealed that Hulunbuir short‐tailed sheep are homozygous CT/CT at nt 333 and 334 in the *TBXT* gene, and Barag sheep also have CT alleles at nt 333 and 334 (Table S1). Although Hulunbuir short‐tailed and Barag sheep are two main strains of Hulunbuir sheep, which belong to the Mongolian sheep group, Chinese fat‐rumped sheep originate mainly from Kazakhstan (Zhong *et al*. 2010; Wei *et al*. 2015). These two main Chinese sheep types were bred with independent selection and evolution. Those findings indicate that c.[333G>C;334G>T] carries strong potential selection for tail length within or among sheep breeds, which may disrupt axial extension of caudal vertebra during early embryo development and finally change the sheep tail type. Therefore, the identification of *TBXT* as a candidate gene may serve as a useful genetic resource for breeding very short‐tailed sheep.

Although the limited samples and statistical methods also could have led to false‐negative findings, owing to the practical difficulties of sample collection, the famous African fat‐rumped Somali and Blackhead Persian sheep breeds could not be included in this study. However, all sheep used in our study categorized as ‘tailless’ carried the dominant genotype—homozygous CT/CT at nt 333 and 334 of *TBXT*—indicating that the mutations contributed to the very short‐tail phenotype in sheep. Our results suggest that the genotype has been completely fixed in the fat‐rumped sheep samples used in the present study. Nonetheless, the genotype was still segregated in fat‐rump‐like Mehraban sheep in Iran, which may be caused by frequent hybridization. Unlike the Iranian Mehraban breed, Chinese purebred fat‐rumped sheep live in a geographically isolated region, where it is almost impossible for them to be crossbred with other breeds. Further studies with extended sampling from African and Middle Eastern sheep will be necessary to understand the genetic diversity of these sheep.

Altogether, our data showed that the c.[333G>C;334G>T] mutation was significantly associated with the tailless phenotype in fat‐rumped sheep. The causative mutations will be a useful genetic resource for future practical studies and also can provide new insights into the breeding plan of long‐tailed sheep for tail shortening by gene modification. Further functional studies in the early embryo will be necessary to elucidate the molecular mechanisms for the devolvement of caudal vertebra underlying these variants in *TBXT*.

## Supporting information


**Table S1** Genotype distribution of *TBXT* c.[333G>C;334G>T] in sheep breeds with different tail types and lengths.
**Table S2** Genotype distribution of *TBXT* c.[333G>C;334G>T] in hybrid offspring populations with different tail types and lengths.
**Table S3** Primers and PCR annealing temperatures used to amplify the coding sequences of the *TBXT* gene.
**Table S4** Genomic variants in the *TBXT* gene identified among the detected sheep populations.
**Figure S1** The number of caudal vertebrae analysis in Kazakh sheep.
