# Supplementary material for: Two linked TBXT (brachyury) gene polymorphisms are associated with the tailless phenotype in fat‐rumped sheep
Source: Anim Genet. 2019 Sep 2;50(6):772–7. doi: 10.1111/age.12852 (PMC6899607; doi:10.1111/age.12852)

**Table S1** Genotype distribution of *TBXT*:c.[333G>C;334G>T] in sheep breeds/ populations with different tail types and lengths.

| **Breeds** | **Sources** | **Phenotype** | | **No.** | **Genotype**(c.333-334G-G> C-T) | | |
| --- | --- | --- | --- | --- | --- | --- | --- |
|  |  | Tail types | Tail lengths |  | C-T/C-T | G-G/C-T | G-G/G-G |
| Kazakh | China | Fat-rumped | Tailless | 36 | 36 | 0 | 0 |
| Altai | China | Fat-rumped | Tailless | 32 | 31 | 1 | 0 |
| Bashbay | China | Fat-rumped | Tailless | 20 | 20 | 0 | 0 |
| Duolang | China | Fat-rumped | Tailless | 30 | 29 | 1 | 0 |
| Bayinbuluke | China | Fat-rumped | Tailless | 33 | 32 | 1 | 0 |
| Cele Black | China | Fat-rumped alike | very short tail | 10 | 0 | 2 | 8 |
| Hulunbuir short tailed sheep | China | Fat-rumped alike | Tailless | 9 | 9 | 0 | 0 |
| Mehraban | Iran | Fat-rumped alike | Tailless or very short tail | 25 | 1 | 7 | 17 |
| Dalagh | Iran | Semi fat-tailed | very short tail | 25 | 24 | 1 | 0 |
| Afshari | ISGC | fat-tailed | Short | 2 | 0 | 1 | 1 |
| Awassi | Israel/ ISGC | Fat-rumped alike | short | 5 | 0 | 0 | 5 |
| Barag sheep | China | Fat-tailed | Short | 7 | 1 | 2 | 4 |
| Mongolian sheep | China | Fat-tailed | Short | 2 | 0 | 0 | 2 |
| Small Tail Han | China | Fat-tailed | Short | 9 | 0 | 0 | 9 |
| Hu | China | Fat-tailed | Short | 23 | 0 | 3 | 20 |
| Tan | China | Fat-tailed | Short | 30 | 0 | 0 | 30 |
| Tibetan | China/ ISGC | Short-tailed | Short | 91 | 0 | 0 | 91 |
| Romanov | American/France | Short-tailed | Short | 13 | 0 | 0 | 13 |
| Beni Guil | morocco | Short-tailed | Short | 6 | 0 | 0 | 6 |
| Merino | Australia/  China/ ISGC | Long-tailed | Long | 46 | 0 | 0 | 46 |
| Alpine Fine-Wool | China | Long-tailed | Long | 32 | 0 | 3 | 29 |
| Alpine Merino | China | Long-tailed | Long | 12 | 0 | 2 | 10 |
| Texel | China | Long-tailed | Long | 32 | 0 | 0 | 32 |
| Dorset | Australia/American | Long-tailed | Long | 36 | 0 | 0 | 36 |
| Katahdin | American | Long-tailed | Long | 7 | 0 | 0 | 7 |
| Dorper | American/ ISGC | Long-tailed | Long | 4 | 0 | 0 | 4 |
| Suffolk | Australia/American | Long-tailed | Long | 16 | 0 | 0 | 16 |
| Rambouillet | American | Long-tailed | Long | 7 | 0 | 0 | 7 |
| D'man | morocco | Long-tailed | Long | 30 | 0 | 0 | 30 |
| Sardi | morocco | Long-tailed | Long | 27 | 0 | 0 | 27 |
| Timahdite | morocco | Long-tailed | Long | 16 | 0 | 0 | 16 |
| Ouled Djellal | morocco | Long-tailed | Long | 8 | 0 | 0 | 8 |
| Total |  |  |  | 681 | 183 | 24 | 474 |

ISGC: international sheep genomics consortium. The samples were obtained from the following Bioprojects. Awassi: 2 from PRJEB12018, 3 from PRJNA160933. Hulunbuir short tail: 8 from PRJNA386449, 1 from PRJNA386274. barag: 7 from PRJNA386449. Mongolian: 2 from PRJNA433439. Duolang: 2 from PRJNA433439. Altay: 2 from PRJNA433439.Afshari: 2 from PRJNA160933. Cele Black: 10 from PRJNA304478. Bayinbuluke: 12 from PRJNA304478. Small Tail Han: 8 from PRJNA304478, 1 from PRJNA357880. Tibetan: 1 from PRJNA357880, 2 from PRJNA433439, 20 from PRJNA281979, 2 from PRJNA160933, 30 from PRJNA304478. Tan: 10 from PRJNA304478, 8 from PRJNA309636. Hu: 9 from PRJNA304478, 2 from PRJNA433439. Merino: 34 from PRJNA325682, 3 from PRJNA160933, 9 from PRJNA304478. Texel: 2 from PRJNA325682, 5 from PRJNA324837, 12 from PRJEB6251, 1 from PRJNA160933. Dorset: 4 from PRJNA436561, 1 from PRJNA33937, 30 from PRJNA325682, 1 from PRJNA160933. Katahdin: 1 from PRJNA222894, 6 from PRJNA324837. Dorper: 2 from PRJNA160933, 2 from PRJNA324837. Suffolk: 14 from PRJNA325682, 2 from PRJNA324837. Romanov: 9 from PRJNA324837, 4 from PRJEB14418; Rambouillet: 3 from PRJNA414087, 4 from PRJNA324837. D'man: 30 from PRJEB3137. Sardi: 27 from PRJEB3137. Timahdite: 16 from PRJEB3137. Beni Guil: 6 from PRJEB3137. Ouled Djellal: 8 from PRJEB3137. Other individuals were sequenced by DNA.PCR production of the Blood

**Table S2** Genotype distribution of *TBXT* c.[333G>C;334G>T] in hybrid offspring populations with different tail types and lengths

| **Populations** | **Phenotype** | | **No.** | **Genotype** (c.333-334 G-G > C-T) | | |
| --- | --- | --- | --- | --- | --- | --- |
|  | Tail types | Tail lengths |  | C-T/C-T | G-G/C-T | G-G/G-G |
| F1a (Kazakh×Tibetan) | Short-tailed | Short | 6 | 0 | 6 | 0 |
| F1b (Kazakh×Texel) | Long-tailed | Long | 6 | 0 | 6 | 0 |
| F2 (F1b ewes backcrossed with Kazakh rams) | Fat-rumped | Tailless | 8 | 8 | 0 | 0 |
|  | Short-tailed | Short | 10 | 0 | 10 | 0 |
| Total |  |  | 30 | 8 | 22 | 0 |

**Table S3** Primers and PCR annealing temperatures used to amplify the coding sequences of the *TBXT* gene.

| Primer name | Sequences | Production | Tm(°C) | Targeted region |
| --- | --- | --- | --- | --- |
| T-1f | GGAAAGTGACAATTCTGCGA | 526bp | 62 | CDs: 1-206 |
| T-1r | GACCTACTCTCGTCCCTCCT |  |  |  |
| T-2f | GCTCTCTGCCACAAGAAGGT | 493bp | 60.5 | CDs: 207-471 |
| T-2r | GCATGCGGATCTAGGTGAGT |  |  |  |
| T-3f | TCCTGTCCTGTTTGGTATGT | 381bp | 60 | CDs: 472-606 |
| T-3r | AAGACATTTTCCTGTGAGCT |  |  |  |
| T-4f | GAAGCTCTGTCTGCTCCATC | 412bp | 61 | CDs: 607-668/669-731 |
| T-4r | GTGGTGCCTGATTTCCTGGT |  |  |  |
| T-5f | CTCAGAGTGGATGAGAGCCA | 212bp | 58 | CDs: 732-789 |
| T-5r | CCACTCCAGTATTCTTGCCT |  |  |  |
| T-6f | CTCTTGAGCCAGTCAAGAGC | 244bp | 60 | CDs: 790-931 |
| T-6r | GCAGCCTGCTTCTCTCACAG |  |  |  |
| T-7f | GGAACTCACGGCAGTTATGT | 420bp | 61 | CDs: 932-1061 |
| T-7r | GGATGACCGATTAAGCAGGT |  |  |  |
| T-8f | GGAGGAGAACTGGAAGCGTG | 793bp | 61 | CDs: 1062-1335 |
| T-8r | ACACAGAAGAAATGAGCCGT |  |  |  |

**Table S4** Genomic variants in the *TBXT* gene identified among the detected sheep populations.

| Location at Oar_v3.1 | Variant ID | cDNA position | Protein position | Alleles | Conseq. Type | Codon change | Amino acids |
| --- | --- | --- | --- | --- | --- | --- | --- |
| 8:87796079 | rs593638946 |  |  | A/G | Downstream gene variant |  |  |
| 8:87796218 | rs408732763 | 1260 | 420 | T/C | Synonymous variant | gcT/gcC | A/A |
| 8:87796223 | rs419979483 | 1255 | 419 | G/A | Missense variant | Gta/Ata | V/I |
| 8:87796266 | rs409523251 | 1212 | 404 | G/A | Synonymous variant | tcG/tcA | S/S |
| 8:87796320 | rs588196058 | 1158 | 386 | A/C | Synonymous variant | cgA/cgC | R/R |
| 8:87796395 | rs596316565 | 1083 | 361 | C/T | Synonymous variant | tcC/tcT | S/S |
| 8:87796427 | rs420796266 |  |  | T/C | Intron 8 variant |  |  |
| 8:87796453 | rs399246531 |  |  | C/T | Intron 8 variant |  |  |
| 8:87796470 | rs410392215 |  |  | T/G | Intron 8 variant |  |  |
| 8:87798792 | rs595980392 |  |  | C/T | Intron 7 variant |  |  |
| 8:87798793 | rs588965102 |  |  | G/C | Intron 7 variant |  |  |
| 8:87798802 | rs414538533 |  |  | G/A | Intron 7 variant |  |  |
| 8:87803383 | rs422002693 |  |  | T/C | Intron 3 variant |  |  |
| 8:87803398 | rs400404465 | 600 | 200 | T/C | Synonymous variant | aaT/aaC | N/N |
| 8:87803410 | rs411641614 | 588 | 196 | T/C | Synonymous variant | acT/acC | T/T |
| 8:87803416 | rs422890370 | 582 | 194 | T/G | Synonymous variant | gcT/gcG | A/A |
| 8:87803540 | rs601835417 |  |  | C/T | Intron 2 variant |  |  |
| 8:87803542 | rs591528020 |  |  | T/C | Intron 2 variant |  |  |
| 8:87803556 | rs401286330 |  |  | T/C | Intron 2 variant |  |  |
| 8:87803583 | rs412508147 |  |  | C/T | Intron 2 variant |  |  |
| 8:87803585 | rs424546372 |  |  | C/T | Intron 2 variant |  |  |
| 8:87804521 | rs594338212 | 402 | 134 | G/A | Synonymous variant | gcG/gcA | A/A |
| 8:87804578 | rs400484411 | 345 | 115 | G/A | Synonymous variant | ccG/ccA | P/P |
| 8:87804589 |  | 334 | 112 | G/T | Missense variant | Ggg/Tgg | G/W |
| 8:87804590 |  | 333 | 111 | G/C | Synonymous variant | ccG/ccC | P/P |
| 8:87804632 | rs407585475 | 291 | 97 | C/T | Synonymous varian | gcC/gcT | A/A |
| 8:87804635 | rs418643399 | 288 | 96 | C/G | Synonymous varian | gcC/gcG | A/A |
| 8:87804668 | rs599863204 | 225 | 85 | C/T | Synonymous varian | aaC/aaT | N/N |
| 8:87804689 | rs401345269 | 234 | 78 | C/T | Synonymous varian | aaC/aaT | N/N |
| 8:87805564 | rs598660281 |  |  | G/- | Upstream gene variant |  |  |
| 8:87805581 | rs604442047 |  |  | G/A | Upstream gene variant |  |  |

**Figure S1** The number of caudal vertebrae analysis in Kazakh sheep.


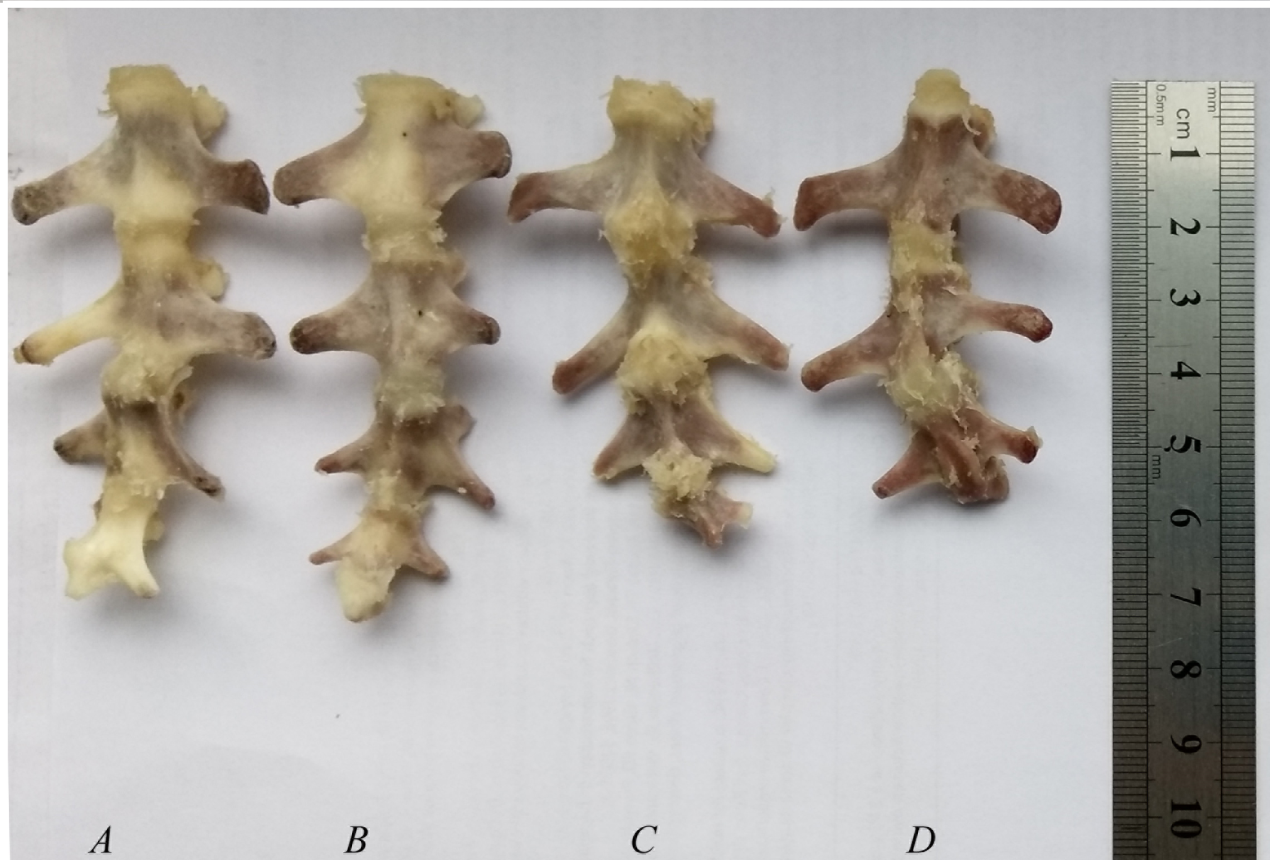

Supplement: Supplementary file 1 — Table S1 Genotype distribution of TBXT c.[333G>C;334G>T] in sheep breeds with different tail types and lengths. Table S2 Genotype distribution of TBXT c.[333G>C;334G>T] in hybrid offspring populations with different tail types and lengths. Table S3 Primers and PCR annealing temperatures used to amplify the coding sequences of the TBXT gene. Table S4 Genomic variants in the TBXT gene identified among the detected sheep populations. Figure S1 The number of caudal vertebrae analysis in Kazakh sheep. [file AGE-50-772-s001.doc]
